# Supplementary material for: Selective formation of acetate intermediate prolongs robust ethylene removal at 0 °C for 15 days
Source: Nat Commun. 2023 May 20;14:2885. doi: 10.1038/s41467-023-38686-0 (PMC10199933; doi:10.1038/s41467-023-38686-0)
Supplement: Supplementary file 1 — Supplementary Information [file 41467_2023_38686_MOESM1_ESM.pdf]

Supplementary Information

Selective formation of acetate intermediate prolongs robust ethylene removal at 0 °C for 15 days

Mingyue Lin[1,2,3], Haifeng Wang[4], Takashi Takei[4], Hiroki Miura[3,4,5], Tetsuya Shishido[3,4,5], Yuhang Li[6], Jinneng Hu[6], Yusuke Inomata[7], Tamao Ishida[4], Masatake Haruta[4], Guangli Xiu[*1], and Toru Murayama[*3,8]

[1]Shanghai Environmental Protection Key Laboratory on Environmental Standard and Risk Management of Chemical Pollutants, State Environmental Protection Key Laboratory of Environmental Risk Assessment and Control on Chemical Process, School of Resources and Environmental Engineering, East China University of Science and Technology, Shanghai 200237, P. R. China

[2]Shanghai Institute of Pollution Control and Ecological Security, Shanghai 200092, P. R. China

[3]Research Center for Hydrogen Energy-based Society, Graduate School of Urban Environmental Sciences, Tokyo Metropolitan University, 1-1 Minami-Osawa, Hachioji, Tokyo 192-0397, Japan

[4]Department of Applied Chemistry for Environment, Graduate School of Urban Environmental Sciences, Tokyo Metropolitan University, 1-1 Minami-Osawa, Hachioji, Tokyo 192-0397, Japan

[5]Elements Strategy Initiative for Catalysts & Batteries, Kyoto University, Kyoto 615-8520, Japan

[6]School of Materials Science and Engineering, East China University of Science and Technology, Shanghai 200237, P. R. China

[7]Faculty of Advanced Science and Technology, Kumamoto University, 2-39-1 Kurokami, Chuo-ku, Kumamoto 860-8555, Japan

[8]Yantai Key Laboratory of Gold Catalysis and Engineering, Shandong Applied Research Center of Gold Nanotechnology (Au-SDARC), School of Chemistry & Chemical Engineering, Yantai University, Yantai 264005, P. R. China

* These authors jointly supervised this work. E-mails: murayama@tmu.ac.jp, xiugl@ecust.edu.cn

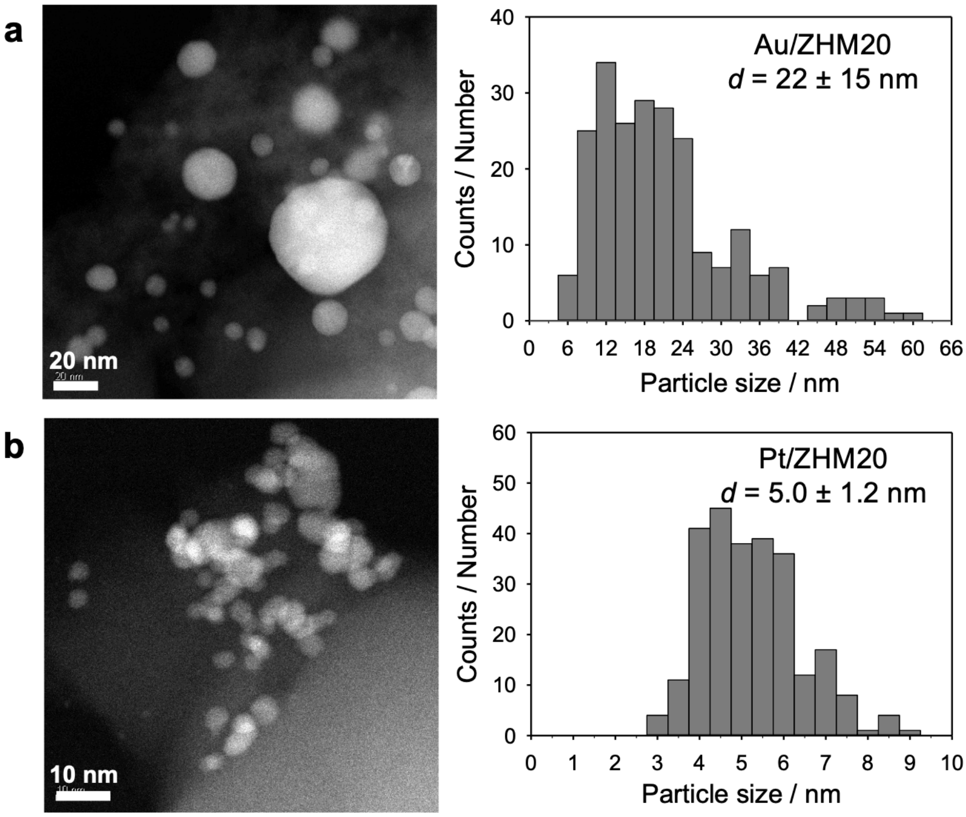

Supplementary Fig. 1 | HAADF-STEM images and size distributions of NPs of (a) Au/ZHM20 and (b) Pt/ZHM20.

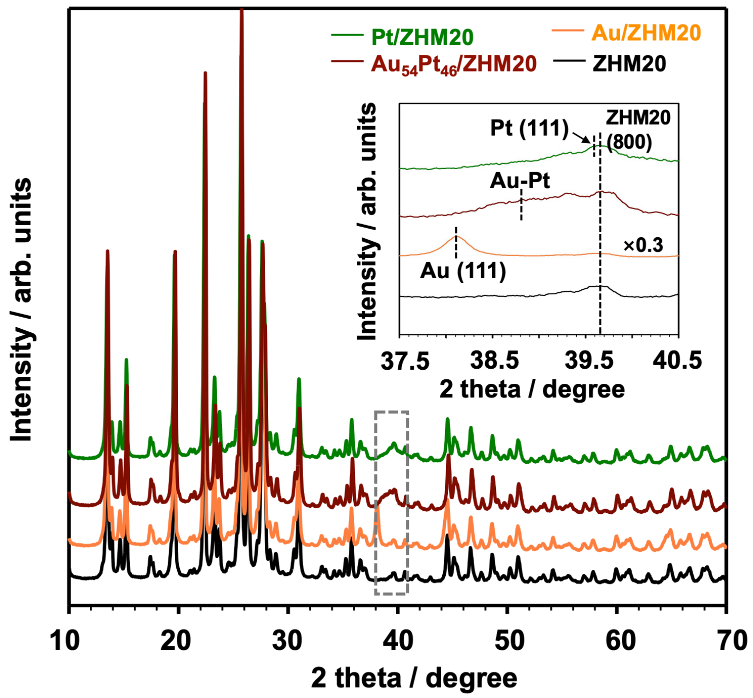

Supplementary Fig. 2 | XRD patterns of ZHM20, Au/ZHM20, $Au_{54}Pt_{46}$/ZHM20, and Pt/ZHM20. arb. units = arbitrary units.

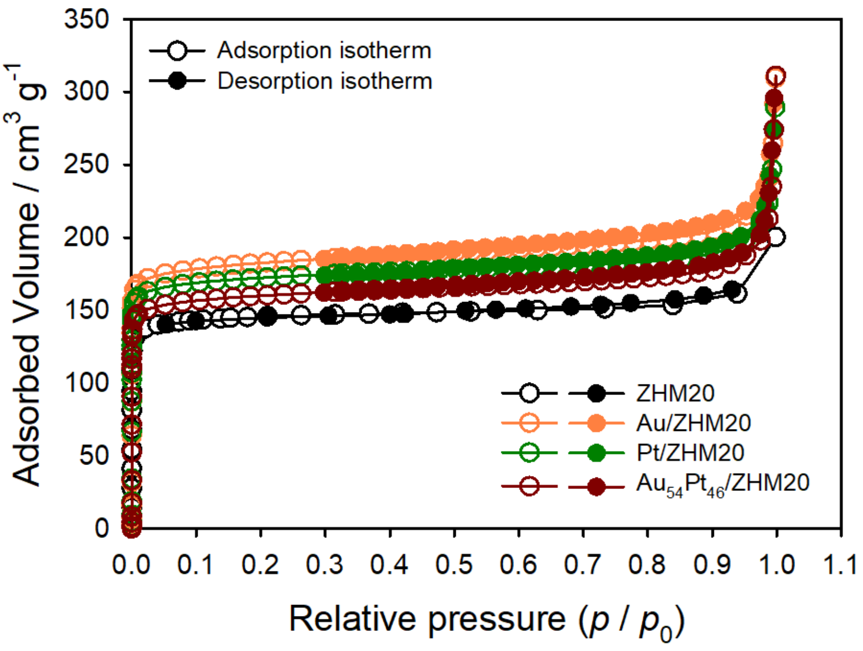

Supplementary Fig. 3 | Nitrogen adsorption and desorption isotherms of ZHM20, Au/ZHM20, Pt/ZHM20, and $Au_{54}Pt_{46}$/ZHM20 measured at 77 K.

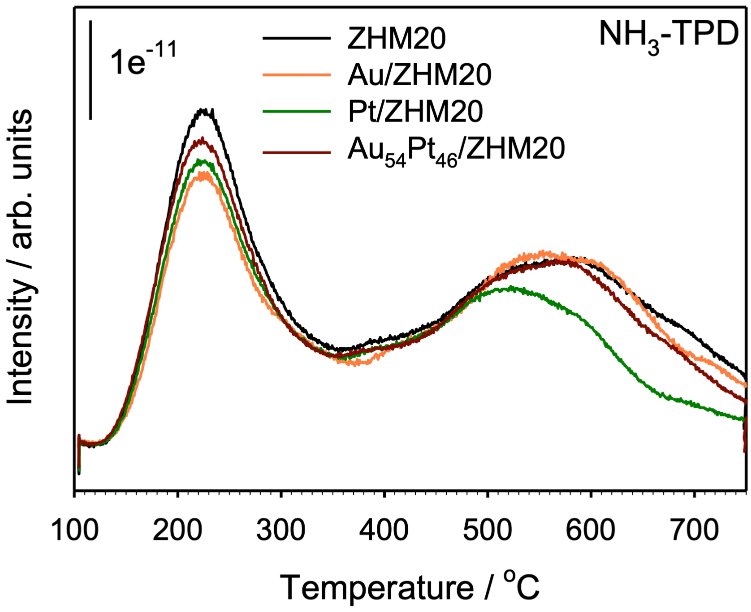

Supplementary Fig. 4 | NH_3-TPD profiles of ZHM20, Au/ZHM20, Pt/ZHM20, and $Au_{54}Pt_{46}$/ZHM20. arb. units = arbitrary units.

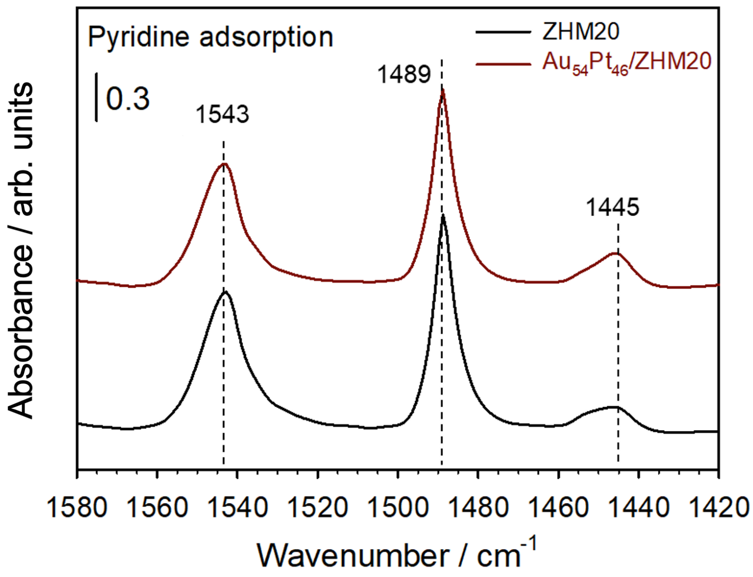

Supplementary Fig. 5 | FT-IR spectra of pyridine adsorbed on ZHM20 and $Au_{54}Pt_{46}$/ZHM20. Pretreatment was carried out under 40 kPa O_2 at 500 °C for 90 min. Pyridine adsorption was conducted at 25 °C for 30 min by introducing 0.4 kPa of pyridine vapor followed by heat treatment at 150 °C for 60 min to remove the physically adsorbed pyridine. The spectrum of chemically adsorbed pyridine on each sample was recorded. arb. units = arbitrary units.

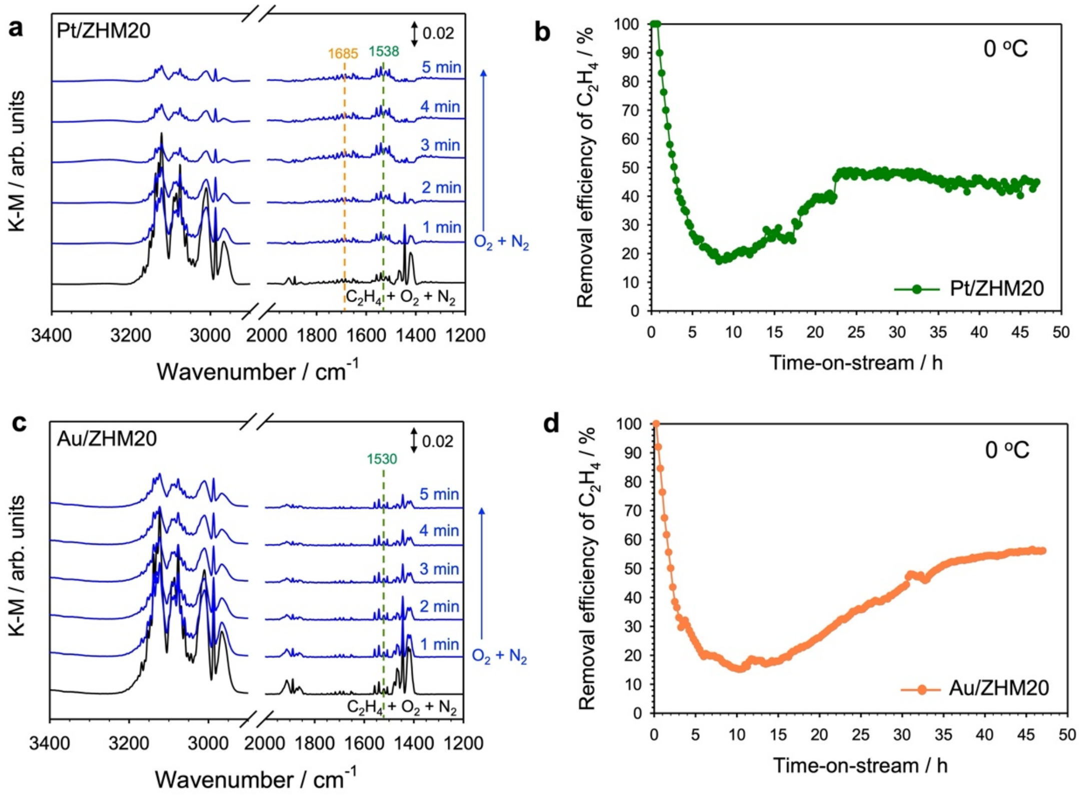

Supplementary Fig. 6 | DRIFT spectra of C_2H_4 oxidation over (a) Pt/ZHM20 and (c) Au/ZHM20 at 0 °C. C_2H_4 removal efficiencies with time-on-stream over (b) Pt/ZHM20 and (d) Au/ZHM20 at 0 °C. DRIFT measurement condition: the sample was pretreated under N_2 flow (50 mL min^{-1}) at 250 °C for 1 h. After cooling to 0 °C, the background spectrum was taken under N_2 flow. Then a mixture of C_2H_4 (25 mL min^{-1}), O_2 (20 mL min^{-1}), and N_2 (55 mL min^{-1}) was flowed for 30 min, and the flow of C_2H_4 was stopped while keeping the flow of O_2 and N_2 for 5 min. Reaction condition: 50 ppm C_2H_4, 20% O_2 and N_2 balance; catalyst, 0.2 g; space velocity, 3000 mL h^{-1} g^{-1}. arb. units = arbitrary units.

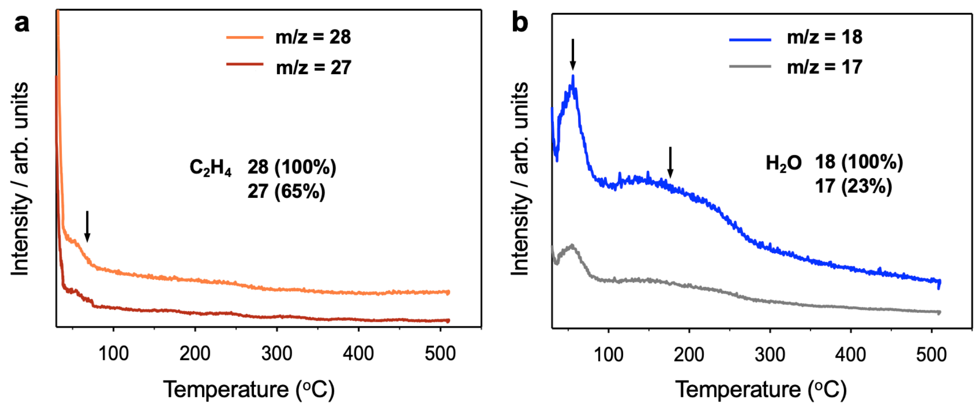

Supplementary Fig. 7 | TPD profiles of (a) C_2H_4 and (b) H_2O of the used $Au_{54}Pt_{46}$/ZHM20. The *x*% represents the theoretical composition of the product by mass. Reaction conditions: C_2H_4 oxidation was firstly carried out on $Au_{54}Pt_{46}$/ZHM20 (0.2 g) at 0 °C for 10 h (81% conversion), and then the used $Au_{54}Pt_{46}$/ZHM20 (0.1 g) was transferred to measure TPD under He flow (30 mL min^{-1}) from 25 °C to 500 °C at a ramp rate of 5 °C min^{-1}. During the desorption, the mass signals of possible products were recorded. arb. units = arbitrary units.

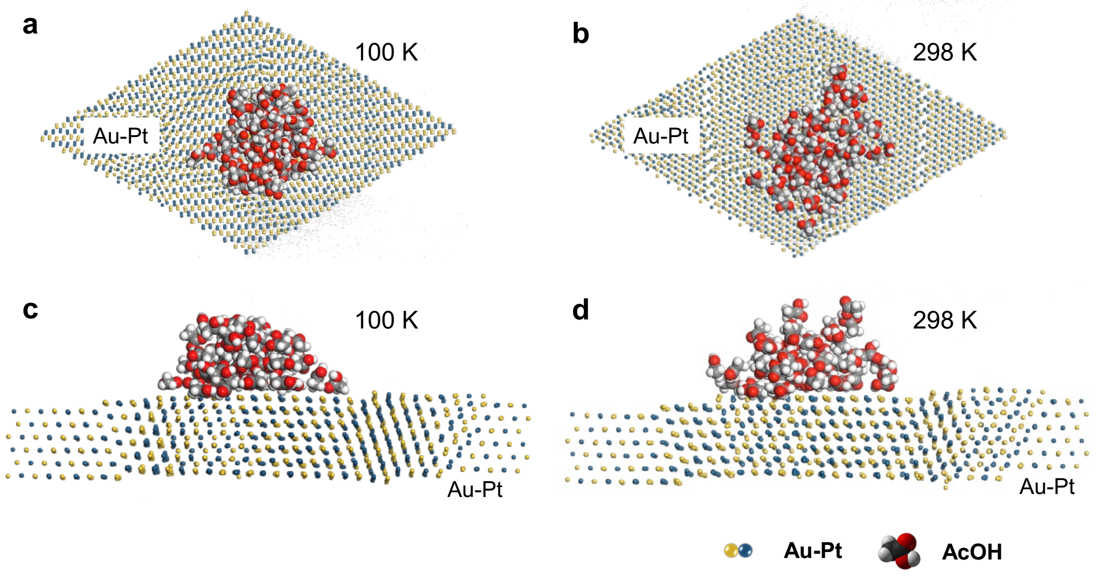

Supplementary Fig. 8 | Top views of Au-Pt alloy nanoparticles after C_2H_4 and AcOH (molar ratio of 50:1) impacts at (a) 100 K and (b) 298 K, and side views of them at (c) 100 K and (d) 298 K. C_2H_4 molecule was included in the simulation but not shown in the figures.

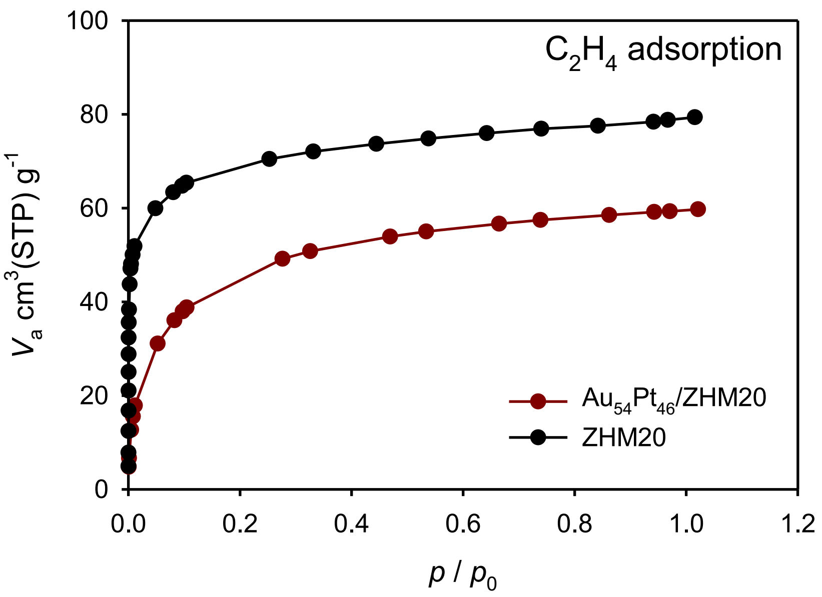

Supplementary Fig. 9 | C_2H_4 adsorption isotherms of $Au_{54}Pt_{46}$/ZHM20 and ZHM20 measured at 0 °C.

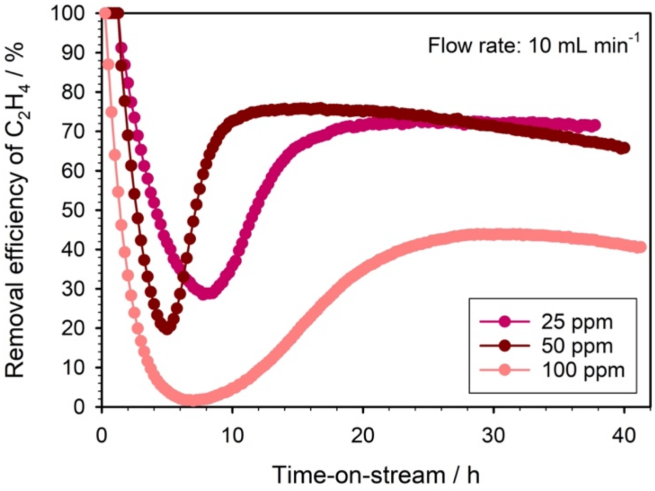

Supplementary Fig. 10 | C_2H_4 removal efficiencies on $Au_{54}Pt_{46}$/ZHM20 at 0 °C with different C_2H_4 concentrations (reaction condition: 25, 50, or 100 ppm C_2H_4, 20% O_2 and N_2 balance; catalyst, 0.2 g).

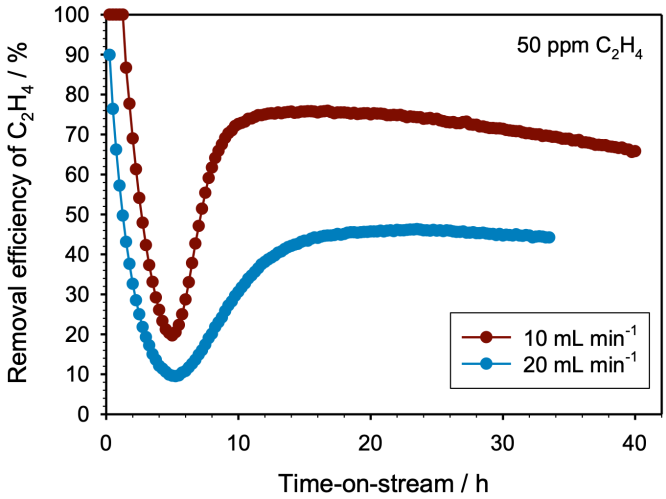

Supplementary Fig. 11 | C_2H_4 removal efficiencies on $Au_{54}Pt_{46}$/ZHM20 at 0 °C with different flow rates (reaction condition: 50 ppm C_2H_4, 20% O_2 and N_2 balance; catalyst, 0.2 g; space velocity, 3000 or 6000 mL h^{-1} g^{-1}).

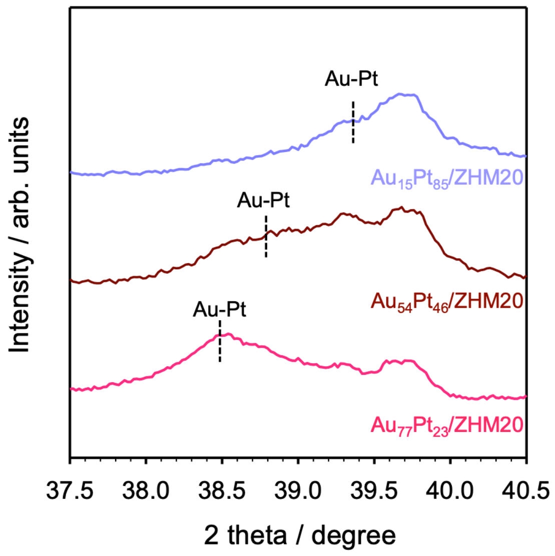

Supplementary Fig. 12 | XRD patterns of $Au_{15}Pt_{85}$/ZHM20, $Au_{54}Pt_{46}$/ZHM20, and $Au_{77}Pt_{23}$/ZHM20. arb. units = arbitrary units.

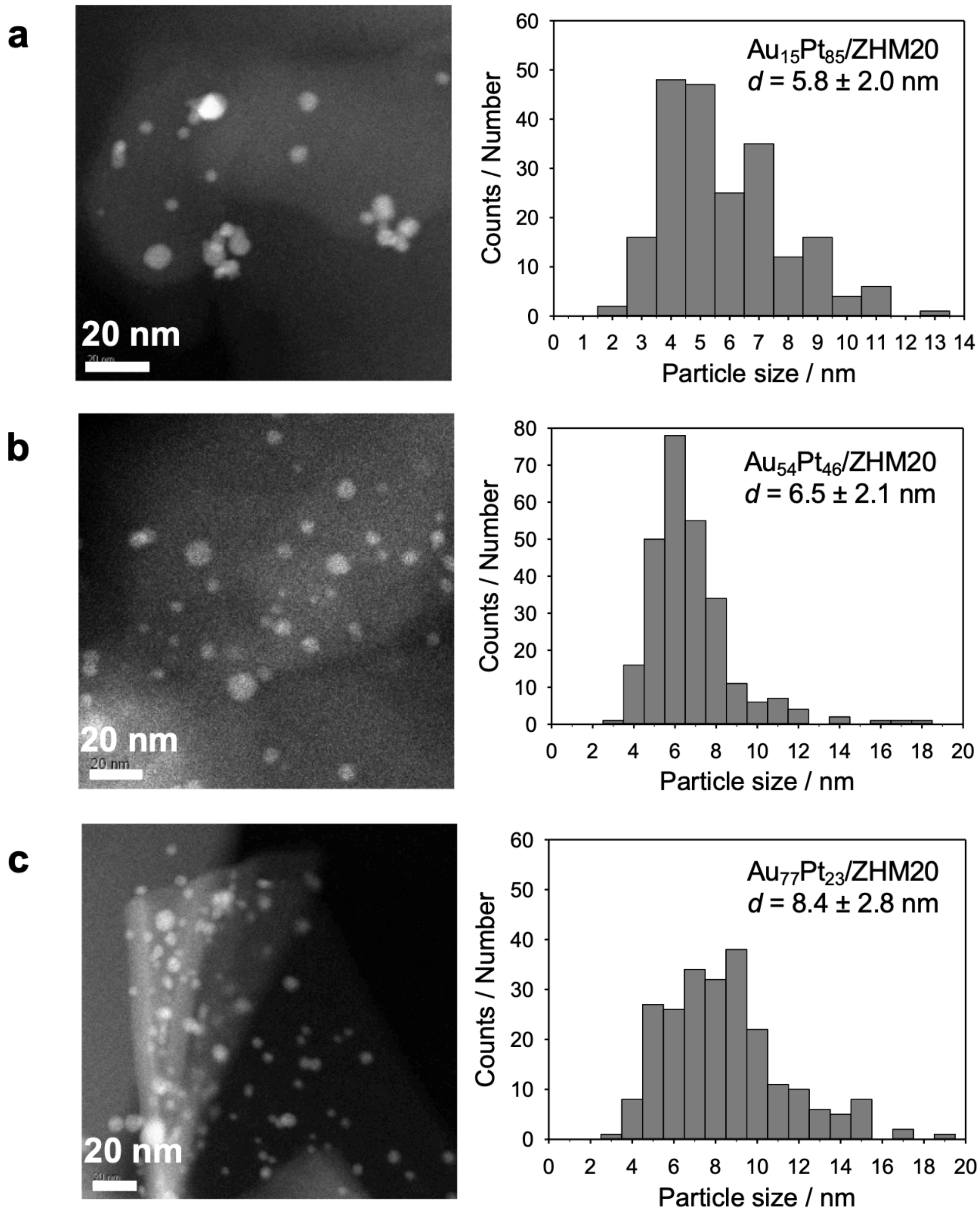

Supplementary Fig. 13 | HAADF-STEM images and size distributions of NPs of (a) $Au_{15}Pt_{85}$/ZHM20, (b) $Au_{54}Pt_{46}$/ZHM20, and (c) $Au_{77}Pt_{23}$/ZHM20.

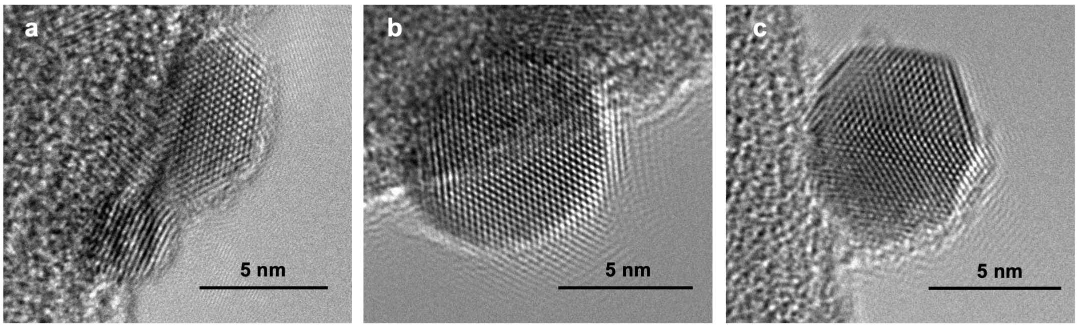

Supplementary Fig. 14 | HRTEM images of (a) $Au_{15}Pt_{85}$/ZHM20, (b) $Au_{54}Pt_{46}$/ZHM20, and (c) $Au_{77}Pt_{23}$/ZHM20.

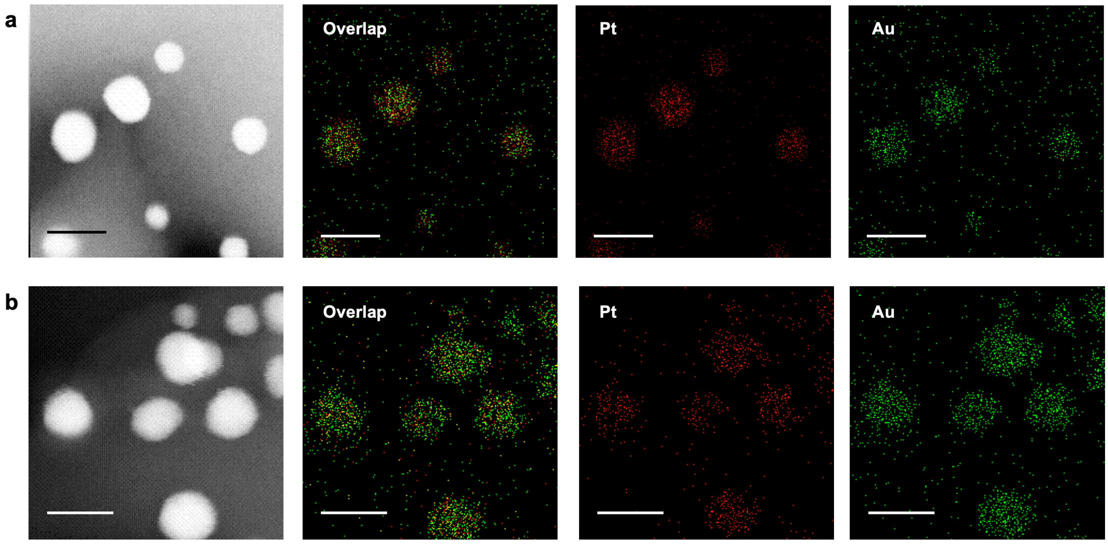

Supplementary Fig. 15 | HAADF-STEM images and corresponding elemental mappings of (a) $Au_{15}Pt_{85}$/ZHM20 and (b) $Au_{77}Pt_{23}$/ZHM20. Scale bar, 25 nm.

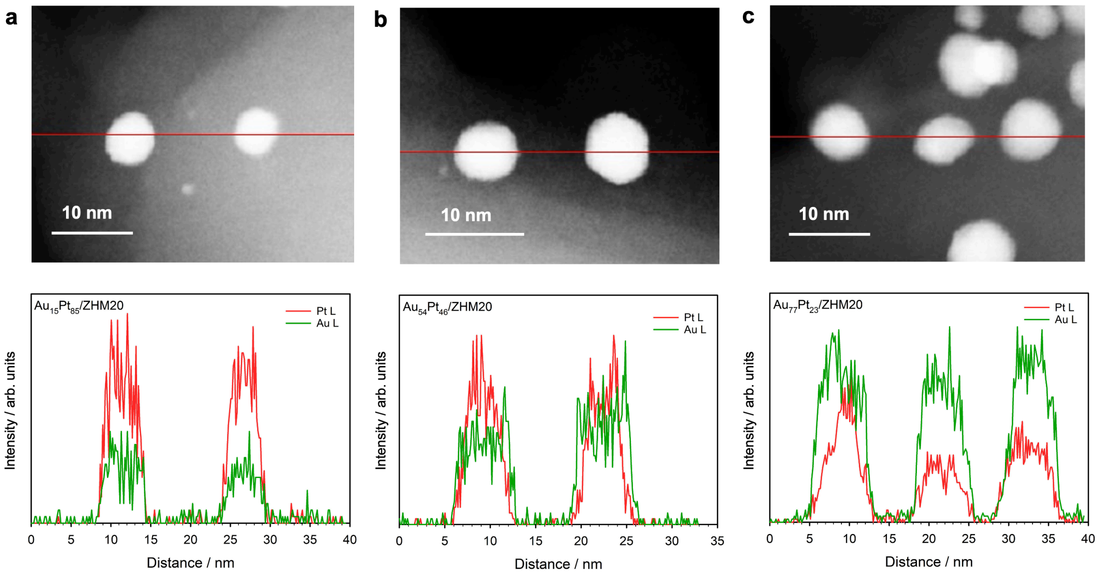

Supplementary Fig. 16 | HAADF-STEM images and corresponding liner analyses of Pt and Au elementals in (a) $Au_{15}Pt_{85}$/ZHM20, (b) $Au_{54}Pt_{46}$/ZHM20, and (c) $Au_{77}Pt_{23}$/ZHM20. arb. units = arbitrary units.

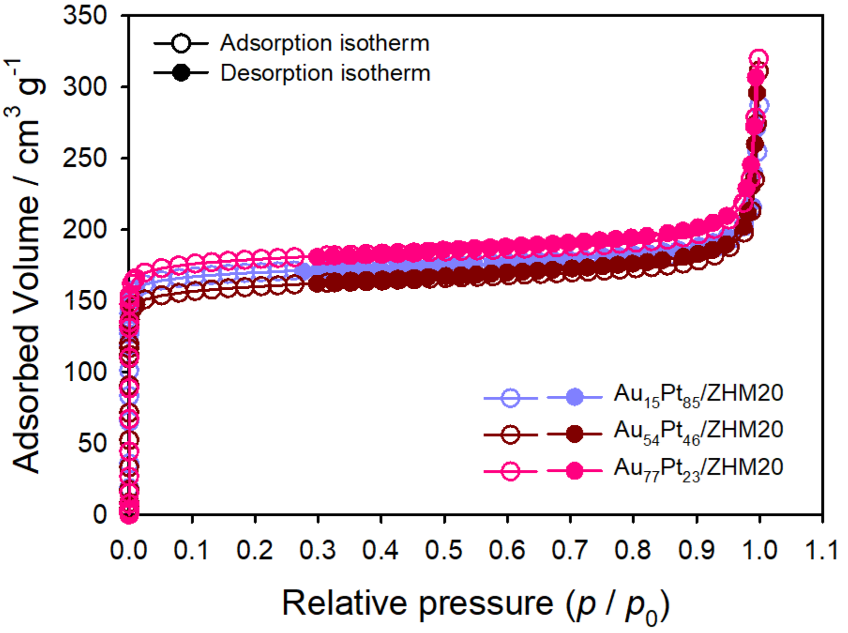

Supplementary Fig. 17 | Nitrogen adsorption and desorption isotherms of $Au_{15}Pt_{85}$/ZHM20, $Au_{54}Pt_{46}$/ZHM20, and $Au_{77}Pt_{23}$/ZHM20 measured at 77 K.

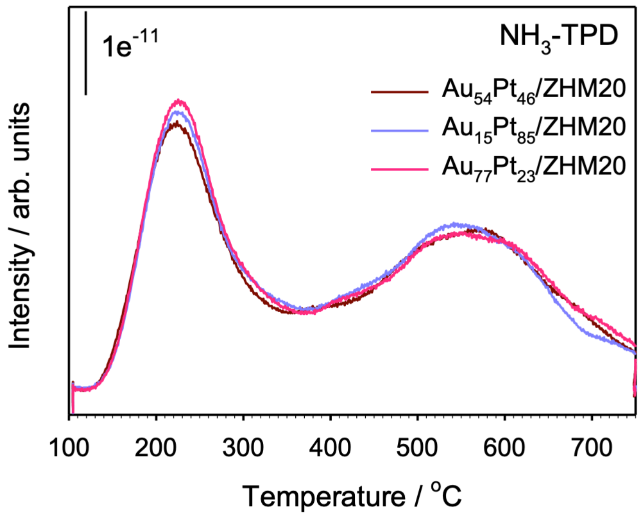

Supplementary Fig. 18 | NH_3-TPD profiles of $Au_{15}Pt_{85}$/ZHM20, $Au_{54}Pt_{46}$/ZHM20, and $Au_{77}Pt_{23}$/ZHM20. arb. units = arbitrary units.

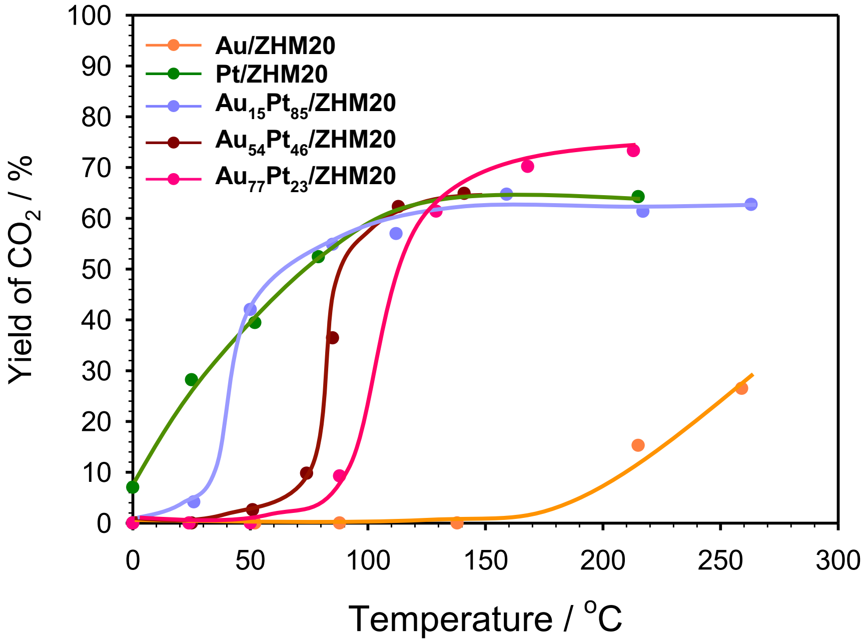

Supplementary Fig. 19 | Temperature dependence of yield of CO_2 during C_2H_4 removal test over Au/ZHM20, Pt/ZHM20, $Au_{15}Pt_{85}$/ZHM20, $Au_{54}Pt_{46}$/ZHM20, and $Au_{77}Pt_{23}$/ZHM20 in the steady state. Conditions: 50 ppm C_2H_4, 20% O_2 and N_2 balance; catalyst, 0.2 g; space velocity, 3000 mL h^{-1} g^{-1}.

Supplementary Table 1 | The prepared and obtained loading amounts of supported metals.

Sample	Prepared (wt%)		Obtained (wt%) [a]		Obtained Au/Pt molar ratio (molar%/molar%)
	Au	Pt	Au	Pt	
Au/ZHM20	1.00	N.A.	0.61	N.A.	100/0
Pt/ZHM20	N.A.	1.00	N.A.	0.34	0/100
$Au_{54}Pt_{46}$/ZHM20	0.50	0.50	0.46	0.39	54/46
$Au_{15}Pt_{85}$/ZHM20	0.09	0.91	0.12	0.66	15/85
$Au_{77}Pt_{23}$/ZHM20	0.80	0.20	0.55	0.16	77/23

[a] Determined by ICP-AES. N.A., not applicable.

Supplementary Table 2 | Physical properties of ZHM20, Au/ZHM20, Pt/ZHM20, and Au-Pt/ZHM20.

Sample	Particle size[a] (nm)	Surface area[b] (m^2/g)	Pore size[b] (nm)	Acid amount[c] (mmol/g)	Ratio of Brønsted/Lewis[d]
ZHM20-AC500	N.A.	835	0.58	1.0	8.7
Au/ZHM20	22 ± 15	766	0.58	1.1	N.A.
Pt/ZHM20	5.0 ± 1.2	828	0.58	0.98	N.A.
$Au_{54}Pt_{46}$/ZHM20	6.5 ± 2.1	826	0.58	0.96	6.0
$Au_{15}Pt_{85}$/ZHM20	5.8 ± 2.0	815	0.58	1.0	5.9
$Au_{77}Pt_{23}$/ZHM20	8.4 ± 2.8	802	0.58	0.97	6.4

[a] Determined by HAADF-STEM. The particle size presents the metal nanoparticles. [b] Calculated by the *t*-plot method. [c] Measured and calculated from NH_3-TPD. [d] Calculated from pyridine adsorption. N.A., not applicable.

Supplementary Table 3 | Comparisons of the C_2H_4 removal efficiency and stability over $Au_{54}Pt_{46}$/ZHM20 with recent reports in literature.

Catalyst	Temperature / °C	C_2H_4 removal efficiency / %	Time-on-stream / h	Total removal[a] / mL	Reference
$Au_{54}Pt_{46}$/ZHM20	0	100 → 2	359	4.40	This study
WTi-Pt2.5	0	100 → 38	2.5[b]	0.096	1
Pt/MCM-41	0	100 → 45	5[b]	0.094	2
Pt/SBA-15	0	99.8 → 55	2[b]	0.049	3
Au/Co_3O_4	0	100 → 98	1[b]	0.001	4
Ag/Beta	25[c]	100 → 0	24	2.58	5
Pt/F-ZSM-5	25[c]	100 → 98	11[b]	1.66	6

[a] The total C_2H_4 removal amount is calculated from the removal curve. [b] Deactivation time was not shown in literature. [c] No C_2H_4 removal results were conducted at around 0 °C in literature.

Supplementary Note 1 | Comparisons of the results under different C_2H_4 concentrations and flow rates for catalytic C_2H_4 removal on $Au_{54}Pt_{46}$/ZHM20 catalyst.

(I) Comparisons of the results under different C_2H_4 concentrations (Supplementary Fig. 10).

- (i) One can see, with the higher concentration of C_2H_4 we applied, the quicker decrease of C_2H_4 removal efficiency in the first several hours. This could reflect the adsorption curve of the ZHM20 support.

- (ii) When the curve reaches the minimum point, the catalytic reaction for converting C_2H_4 may start and the removal efficiency increases again, thus showing a U-shape curve.

- (iii) We noted, with a low C_2H_4 concentration of 25 ppm, that the catalyst shows a delay activation than that of 50 ppm C_2H_4, suggesting the transport limitation under the condition of 25 ppm C_2H_4, in which the catalytic reaction is slower than that of 50 ppm C_2H_4.

- (iv) The similar removal efficiency in the steady state under conditions of 25 and 50 ppm C_2H_4 suggests the catalyst may have a maximum removal efficiency of ~80% under the flow rate of 10 mL min^{-1}. This may also be due to the transport limitation.

- (v) We further found, in the high C_2H_4 concentration case (100 ppm C_2H_4), that the removal efficiency is about half of that of 50 ppm C_2H_4 (**Supplementary Fig. 11**). This suggests a similar catalytic rate in both conditions.

(II) Comparisons of the results under different flow rates (Supplementary Fig. 11).

- (i) When we increased the flow rate in C_2H_4 removal experiments to 20 mL min^{-1}, in which the space velocity is 6000 mL h^{-1} g^{-1}, we can observe, with the higher flow rate we set, the quicker decrease of C_2H_4 removal efficiency is observed in the first several hours. This could reflect the adsorption curve of the ZHM20 support.

- (ii) We found the C_2H_4 removal rates are similar at both flow rates, indicating the flow rate may have negligible influence on the activity of $Au_{54}Pt_{46}$/ZHM20 catalyst.

Supplementary References

1. Guo, H. *et al.* Hierarchical porous wood cellulose scaffold with atomically dispersed Pt catalysts for low-temperature ethylene decomposition. *ACS Nano*, **13**, 14337-14347 (2019).
2. Satter, S. S., Hirayama, J., Kobayashi, H., Nakajima, K. & Fukuoka, A. Water-resistant Pt sites in hydrophobic mesopores effective for low-temperature ethylene oxidation. *ACS Catal.*, **10**, 13257-13268 (2020).
3. Jiang, C., Hara, K. & Fukuoka, A. Low-temperature oxidation of ethylene over platinum nanoparticles supported on mesoporous silica. *Angew. Chem. Int. Ed.*, **52**, 6265-6268 (2013).
4. Li, J. *et al.* Efficient elimination of trace ethylene over nano-gold catalyst under ambient conditions. *Environ. Sci. Technol.*, **42**, 8947-8951 (2008).
5. Yang, H. *et al.* Understanding the active sites of Ag/zeolites and deactivation mechanism of ethylene catalytic oxidation at room temperature. *ACS Catal.*, **8**, 1248-1258 (2018).
6. Yang, H. *et al.* Fluorine-enhanced Pt/ZSM-5 catalysts for low-temperature oxidation of ethylene. *Catal. Sci. Technol.*, **8**, 1988-1996 (2018).
